# Differential roles of gangliosides in malignant properties of melanomas

**DOI:** 10.1371/journal.pone.0206881

**Published:** 2018-11-21

**Authors:** Yuhsuke Ohmi, Mariko Kambe, Yuki Ohkawa, Kazunori Hamamura, Orie Tajima, Rika Takeuchi, Koichi Furukawa, Keiko Furukawa

**Affiliations:** 1 Department of Clinical Engineering, Chubu University College of Life and Health Sciences, Kasugai, Aichi, Japan; 2 Department of Biomedical Sciences, Chubu University College of Life and Health Sciences, Kasugai, Aichi, Japan; 3 Department of Pharmacology, School of Dentistry, Aichigakuin University, Nagoya, Japan; 4 Department of Biochemistry II, Nagoya University Graduate School of Medicine, Nagoya, Japan; Duke University School of Medicine, UNITED STATES

## Abstract

Ganglioside GD3 is widely expressed in human malignant melanomas, and has been reported to be involved in the increased cell proliferation and invasion. In this study, we established GM3-, GM2-, GM1-, GD3-, or GD2-expressing melanoma cell lines by transfecting cDNAs of glyscosyltransferases, and effects of individual gangliosides on the cell phenotypes and signals were examined. The phenotypes of established ganglioside-expressing cells were quite different, i.e. cell growth increased as following order; GD2+, GD3+ > GM1+, GM2+, GM3+ cells. Cell invasion activity increased as GD3+ ≧ GM2+ > GM1+, GM3+, GD2+ cells. Intensity of cell adhesion to collagen I (CL-I) and spreading increased as GD2+ >> GD3+, GM1+ > GM2+, GM3+ cells. In particular, cell adhesion of GD2+ cells was markedly strong. As for cell migration velocity, GD2+ cells were slower than all other cells. The immunocytostaining revealed close localization of gangliosides and F-actin in lamellipodia. Immunoblotting of phosphorylated p130Cas and paxillin by serum treatment reveled that these phosphorylations were more increased in GD3+ cells than in GD2+ or GM3+ cells, while phosphorylation of Akt underwent similarly increased phosphorylation between GD3+ and GD2+ cells compared with GM3+ cells. While GD2 and GD3 enhanced cell growth, GD3 might also contribute in cell invasion. On the other hand, GD2 might contribute in the solid fixation of melanoma cells at metastasized sites. These results suggested that individual gangliosides exert distinct roles in the different aspects of melanomas by differentially regulating cytoskeletons and signaling molecules.

## Introduction

Sialic acid-containing glycosphingolipids are highly expressed in nervous tissues of mammals and birds [1], and have been considered to be involved in the development and function of nervous systems [2,3]. Recent advances in the functional analysis of gangliosides using genetically modified experimental animals revealed that gangliosides play pivotal roles in the maintenance and repair of nervous tissues [4–6]. In turn, gangliosides with relatively simple structures have been identified as cancer-associated antigens, since they are specifically expressed in cancer tissues in neurocrest-derived cancers and some leukemia cells [7–10]. Therefore, they have been used as tumor markers [11,12], and as targets of antibody therapy in melanomas [13] and neuroblastomas [14,15].

Since we isolated cDNA clones of gangliosides GM2/GD2 synthase (*B4GALNT1*)[16], we have isolated cDNAs of various glycosyltransferases involved in the synthesis of gangliosides, i.e. GD3 synthase (*ST8SIA1*)[17], GM1/GD1b/GA1 synthase (*B3GALT4*) [18], GM3 syntase (*ST3GAL5*) [19], GD1α synthase (*ST6GALNT5*) [20] and so on, and analyzed functions of their products in the maintenance of homeostasis of normal tissues [4,21], and in the positive/negative regulation of malignant properties of cancer cells [22].

Depending on the fine structures of individual gangliosides, characteristic effects on the phenotypes of cells and cell signaling have been observed. In particular, expression and roles of disialylated gangliosides such as GD3 and GD2 in various malignant tumors have been rigorously investigated, since GD3/GD2 was identified as melanoma-associated gangliosides [23–27]. They were frequently recognized by immune systems of patients [23,27]. The facts that anti-GD3 monoclonal antibody could induce reduction of metastatic melanomas when injected into patients [13] strongly prompted us to examine the function of disialyl gangliosides, GD3/GD2. Actually, we have analyzed their enhancing activity of signals involved in the malignant properties of cancer cells in small cell lung cancers [28], malignant melanomas [29], and osteosarcomas [30]. On the other hand, monosialylated ganglioside GM1 suppressed cell signals in NGF-induced differentiation of PC12 cells [31]. Transfection of GM1 synthase also suppressed malignant properties of a melanoma cell line [32]. Suppression of EGF/EGF receptor signals by GM3 was reported by other group [33]. Consequently, opposite effects on cancer properties between disialylated gangliosides and monosialylated gangliosides have been broadly recognized [22].

Thus, different ganglioside structures appear to exert distinct roles in cancer cells, leading to different directions of cell phenotypes. Here, we analyzed implication of representative gangliosides in phenotypes of melanoma cells using various transfectant cells with glycolipids remodeling on the same genetic background, and demonstrated differential effects on various features of melanoma cells. In particular, disialyl gangliosides GD3 and GD2 showed distinct actions suggesting their individual roles in different aspects of melanomagenesis and expansion.

## Materials and methods

### Cell culture and preparation of transfectant cells expressing individual gangliosides

GD3-expressing N1 cells (GD3+ cells) were established from a GD3-non-expressing mutant of SK-MEL-28, N1 (N1 cells) [29,34] by transfection of human GD3 synthase cDNA [17]. N1 cells transfected with a vector alone were established as controls (GM3+ control cells). GM2-expressing N1 cells (GM2+ cells) were established by transfection of human GM2 synthase cDNA [16] into N1 cells as GD3+ cells. GM1-expressing N1 cells (GM1+ cells) were prepared by transfection of GM1 synthase cDNA [18] into GM2-expressing N1 cells. These cells were established with 400 μg/mL of G418. GD2-expressing (GD2+ cells) cells were also established by transfection of human GM2 synthase cDNA into GD3-expressing cells with 600 μg/mL of G418. These cells were maintained in Dulbecco’s modified Eagle’s medium (D-MEM) supplemented with 7.5% FCS and G418 (400 μg/mL or 600 μg/mL) at 37°C in a humidified atmosphere containing 5% CO_2_.

### Antibodies

Anti-GM3 mouse monoclonal antibody (mAb) was purchased from NIPPON BIO-TEST LABORATORIES INC. (Saitama, Japan). Anti-GD3 mAb R24 and anti-GM2 mAb 10–11 were kindly provided from Dr. Lloyd J. Old (Memorial Sloan-Kettering Cancer Center) and from Dr. P. O. Livingston, respectively. Anti-GD1a mAb D226 and anti-GD2 mAb 220–51 were previously generated in our laboratory [35]. GM1 was detected by Cholera Toxin B subunit biotin conjugate purchased from Sigma-Aldrich Co. (Saint Louis, MO) and Streptavidin-FITC Conjugate from Thermo Fisher Scientific (Waltham, MA). All these mAbs are specific to individual gangliosides. Anti-rabbit IgG antibody conjugated with horseradish peroxidase (HRP) and Anti-mouse IgG antibody conjugated with HRP were purchased from Cell Signaling Technology (Beverly, MA). Anti-p130Cas antibody was purchased from BD Transduction Laboratories (San Jose, CA) and anti-phospho-p130Cas antibodies (Tyr-165, Tyr-249, and Tyr-410) were from Cell Signaling Technology. Anti-paxillin antibody was purchased from BD Transduction Laboratories. Anti-phospho-paxillin antibodies (Tyr-31 and Tyr-181) were purchased from Santa Cruz Biotechnology (Santa Cruz, CA) and anti-phospho-paxillin antibody (Tyr-118) was from Cell Signaling Technology. Anti-Akt antibody, anti-phospho-Akt (Ser-473) antibody and anti-phospho-Akt (Thr-308) mAb were purchased from Cell Signaling Technology. Anti-ß-Actin mAb was purchased from Cell Signaling Technology. Anti-phosphotyrosine antibody (PY20) was purchased from Transduction Laboratories (Lexington, KY).

### Reagents

Collagen type I (CL-I) (Merck Millipore, Burlington, MA), Collagen type IV (CL-IV) (Chemicon, Temecula, CA) and Fibronectin (FN) (Chemicon, Temecula, CA) were used for coating plates. Culture plates or glass-base dishes were coated by CL-1, CL-IV or FN at 5 μg/mL in PBS at room temperature for 1 h, and blocked by 1% BSA/D-MEM at room temperature for 1 h. Actin filament was stained by CytoPainter Phalloidin-iFluor 488 (Abcam, Cambridge, UK).

### Cell proliferation assay

Cell proliferation was measured by Thiazolyl Blue tetrazolium Bromide (MTT) (5mg/mL in PBS) as described [36]. Briefly, Cells were plated after trypsinization into 96-well plates at 3×10^3^ cells/100μL/well, and incubated at 37°C in CO_2_ incubator. MTT solution (20 μL of 5 mg/mL) was added into each well and incubated for 4 h. After confirming of the formation of needle-like crystals, acidic propylalcohol (150 μL/well) was added. Hundred μL of supernatants was transferred after pipetting into another plate, and absorption of each well was measured using a plate reader (TECAN, Männedorf, Switzerland) at 570 nm with reference at 650 nm.

### Cell adhesion and expansion as measured by RT-CES

Real-time cell electronic sensing system (RT-CES) (Wako Pure Chemical, Osaka, Japan), was used for analysis of cell adhesion and expansion. Changes in the biological status of cells can be measured automatically in real time by RT-CES. Microelectronic cell sensor arrays are integrated into the bottom of the micro-plates (E-Plate (16X) (ACEA Biosciences Inc., San Diego, CA)). This sensor provides continuous and quantitative information concerning the biological status of the cells in the well and the increased electrical resistance (Cell Index) indicates the increase of cell adhesion and expansion. E-Plate was coated by CL-1, CL-IV or FN at 5 μg/mL in PBS (100 μL/well) at room temperature for 1 h, and blocked by 1% BSA/D-MEM (100 μL/well) at room temperature for 1 h. After blocking of the plate, cells (1 x 10^4^) were added in each well of the plate containing culture medium and changes in cell adhesion and expansion were monitored continuously.

### Flow cytometry (FCM)

Surface expression of gangliosides was analyzed by FCM with Accuri C6 Flow Cytometer (BD Biosciences, Bedford, MD or BD, Franklin Lakes, NJ). Monoclonal antibodies used for detection of individual ganglisoides were as follows, anti-GD3, R24 (mouse IgG3); anti-GD2, 220–51 (mouse IgG1); anti-GD1a, D266 (mouse IgM); anti-GM2, 10–11 (mouse IgM); anti-GM3, M2590 (mouse IgM). GM1 was detected by biotin-choleratoxin-B combined with streptavidin-FITC (ZYMED, Carlsbad, CA). After incubation of cells with individual antibodies for 1 h on ice, cells were washed twice with PBS. Then, secondary antibodies (anti-mouse IgG H&L) were added, and incubated for 45 min on ice. Fluorescence intensity of cells was analyzed with Accuri C6 using controls prepared by normal mouse Igs with same isoforms.

### Cell invasion assay

Cell invasion was analyzed by Boyden-chamber (8.0 μm pore size) method with Matrigel as described previously [29]. FCS was not added in the low chamber.

### Cell mobility

Cells were added to the glass-base dish precoated with CL-1, and time-lapse observation was started after 30 min by the confocal laser scaning microscope (FV10i, Olympus). The time-lapse imaging was recorded every 10 min for 20 h. Then, cell migration was analyzed by FLUOVIEW software for FV10i. The position of each cell was plotted and traces of cell migration were drawn. Migration distances of cells were calculated by Pythagoras theorem using X and Y coordinates, and relative migration velocity was obtained as migration distance/min.

### Immunofluorescence assay

Cells were cultured in CL-I-precoated dishes for 1 h and fixed by 4% paraformaldehyde. GD3 and GD2 were stained with anti-GD3 mAb (R24) and anti-GD2 mAb (220–51). Anti-mouse IgG-Alexa-594 (red) (Thermo Fisher Scientific, Waltham, MA) was used as a secondary antibody. F-actins were stained with CytoPainter Phalloidin-iFluor 488 reagents (green). GM2 and GM3 were also stained with anti-GM2 mAb (10–11) and anti-GM3 mAb (M2590). Anti-mouse IgM-Alexa594 (red) (Jackson ImmunoReserch. inc., West Grove, PA) was used as a secondary Ab.

### Preparation of cell lysates

Cells were lysed with cell lysis buffer (20 mM Tris-HCl, 150 mM NaCl, 1 mM Na_2_EDTA, 1 mM EGTA, 1% Triton X-100, 2.5 mM sodium pyrophosphate, 1 mM ß-glycerophosphate, 1 mM Na_3_VO_4_, 1 mM leupeptin) (Cell Signaling Technology) added with 1 mM PMSF. Insoluble materials were removed by centrifugation at 10,000 x *g* for 10 min at 4°C.

### Western immunoblotting

Cell lysates were separated by SDS-PAGE using 8~10% gels. The separated proteins were transferred onto an Immunobilon-P membrane (Millipore, Billerica, MA). Blots were blocked with 3% BSA in PBS containing 0.05% Tween 20. The membrane was first probed with primary antibodies. After being washed, the blots were incubated with anti-mouse or rabbit IgG conjugated with HRP. After washing, bound conjugates on the membrane were visualized with an Enhanced Chemiluminescence detection system (PerkinElmer Life Sciences, Waltham, MA) or ImmunoStar LD (Wako Pure Chemical, Osaka, Japan). Chemiluminescence was detected and analyzed by Amersham Imager 680 (GE Healthcare UK Ltd, Buckinghamshire, UK). Chemiluminescence for PY20 was detected by Super RX fuji medical X-ray film (Fuji film). Signal intensity was analyzed by Image J software [37].

### Knockdown for *hB4GALNT1* gene in GD2+ cells

*hB4GALNT1*-specific siRNA or control siRNA (MISSION siRNA Universal Negative Control) were purchased from Sigma-Aldrich. These siRNAs (50 pmol) were transfected into 1 × 10^5^ cells/35 mm dish of GD2+ cells by Lipofectamine RNAiMAX (ThermoFisher). siRNA-transfected cells were incubated for 3 day or 1 week to perform real time RT-PCR or flow cytometry and invasion assay, respectively.

### Real-time reverse transcription-polymerase chain reaction (RT-PCR)

Total RNA was extracted from cell lines with RNeasy Plus Mini kit (QIAGEN) according to the manufacturer’s protocol and then reverse-transcribed into cDNA by using M-MLV Reverse Transcriptase (ThermoFisher) and oligo dT primer (Sigma-Aldrich). Real-time RT-PCR was performed using 1 ng of RNA/cDNA per well, with SsoAdvanced Universal SYBR green Supermix (Bio-Rad Laboratories, Inc., Hercules, CA), and Thermal Cycler CFX Connect (Bio-Rad Laboratories). The PCR conditions were as follows: preheating for 3 min at 95°C, 40 cycles of 95°C (10 s), 58°C (10 s), and 72°C (20 s). The signal reader was set at 72°C depending on individual primer pairs. *hGAPDH* gene expression was used as a standard. Every sample was measured in duplicate, and gene expression levels were analyzed by using CFX Manager 2.1 software (Bio-Rad Laboratories).

### Statistical analysis

Data were presented as means ± standard deviation (SD) in individual experiments. Results were initially analyzed for homogeneity of variance using Bartlett’s, Hartley’s, and Levene’s tests. The Shapiro-Wilk test was used to verify that the data followed normal distribution. Statistical significance was calculated by using two-tailed Student’s t test. All statistical significances were set as *, *p* <0.05; **, *p* <0.01; ***, *p* <0.001.

## Results

### Establishment of transfectant cells mainly expressing single major gangliosides

**Fig 1:**
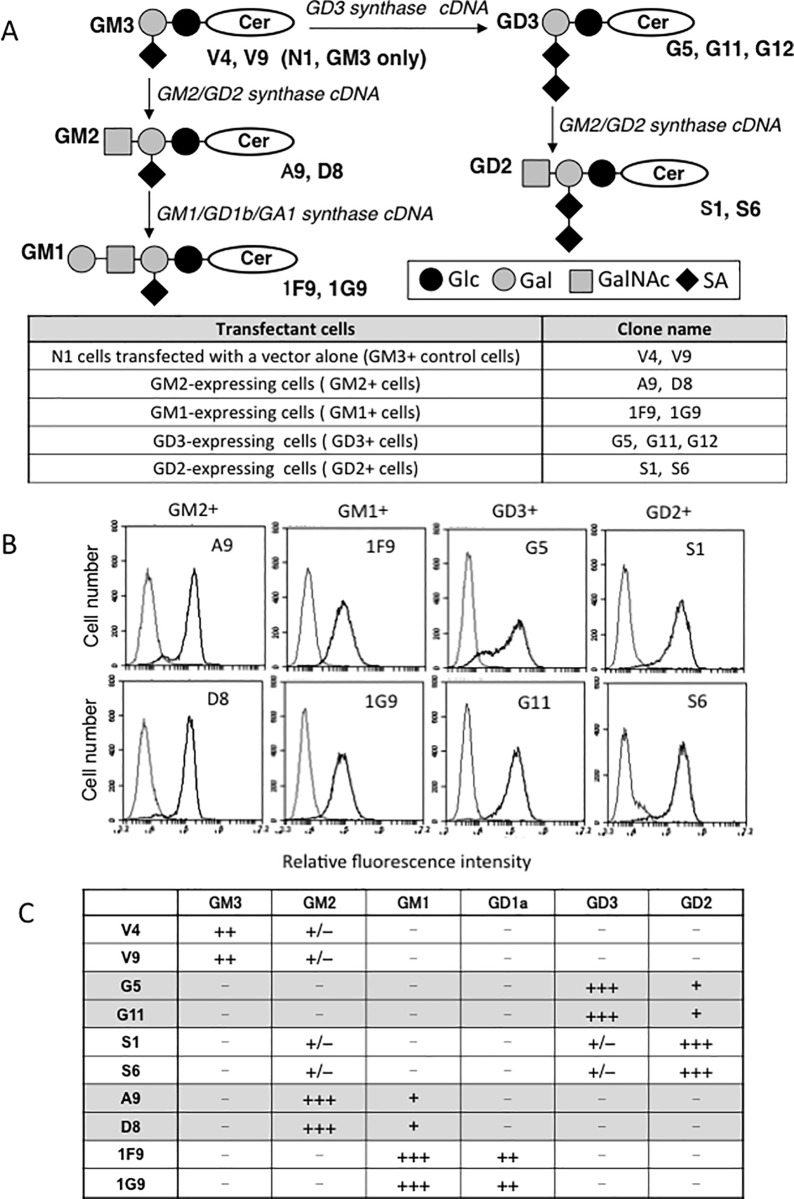
Establishment of various ganglioside-expressing cells. (A) Biosynthetic pathway of gangliosides and glycolsyltransferase cDNAs used to establish various ganglioside-expressing cells. (B) Flow cytometry of ganglioside expression on GM2-, GM1-, GD3-, and GD2-expressing transfectans. GM2, GD3, and GD2 were detected with mAb 10–11, mAb R24, and mAb 220–51, respectively. GM1 was detected with biotin-choleratoxin-B. (C) Summary of ganglioside expression on transfectant cells. Depending on the ratios of positive cells, results of flow cytometry were scored from–, +, ++, and +++. *O*-acetylated gangliosides such as 9-*O*-acetyl GD3 have been also paid attention for their unique expression and function [44], though they were not detected as major species here.

A subline of a melanoma cell line, SK-MEL-28 N1 was transfected by cDNAs of GM2/GD2 synthase, GD3 synthase, combination of GM2/GD2 synthase and GD3 synthase, or GM2/GD2 synthase and GM1 synthase as shown in Fig 1A (*upper*). N1 cells expressed GM3 almost exclusively [34]. Transfectants with vector alone were designated V4 and V9. GM2-expressing cells were named A9 and D8. GD3-expressing cells were G5 and G11. GD2-expressin cells were S1 and S6, and GM1-expressing cells were 1F9 and 1G9. The nomenclature for cells expressing different gangliosides was indicated in Fig 1A (*lower*). These cells almost exclusively expressed individual gangliosides except 1F9 and 1G9, in which GD1a was expressed as well as GM1. Represenative FCM patterns were shown in Fig 1B, and Expression profiles of gangliosides in these transfectant cells were summarized in Fig 1C by citing our previous results [38] with modification.

The cell surface expression of gangliosides as analyzed by FCM is important for interactions with extracellular molecules. Total gangliosides in these transfectant cells as analyzed by thin layer chromatography (TLC) were already reported in our previous paper (S1 Fig of [38]), and major gangliosides detected in TLC well corresponded with those found by FCM.

### GD3+ cells and GD2+ cells showed increased cell growth in MTT assay

**Fig 2:**
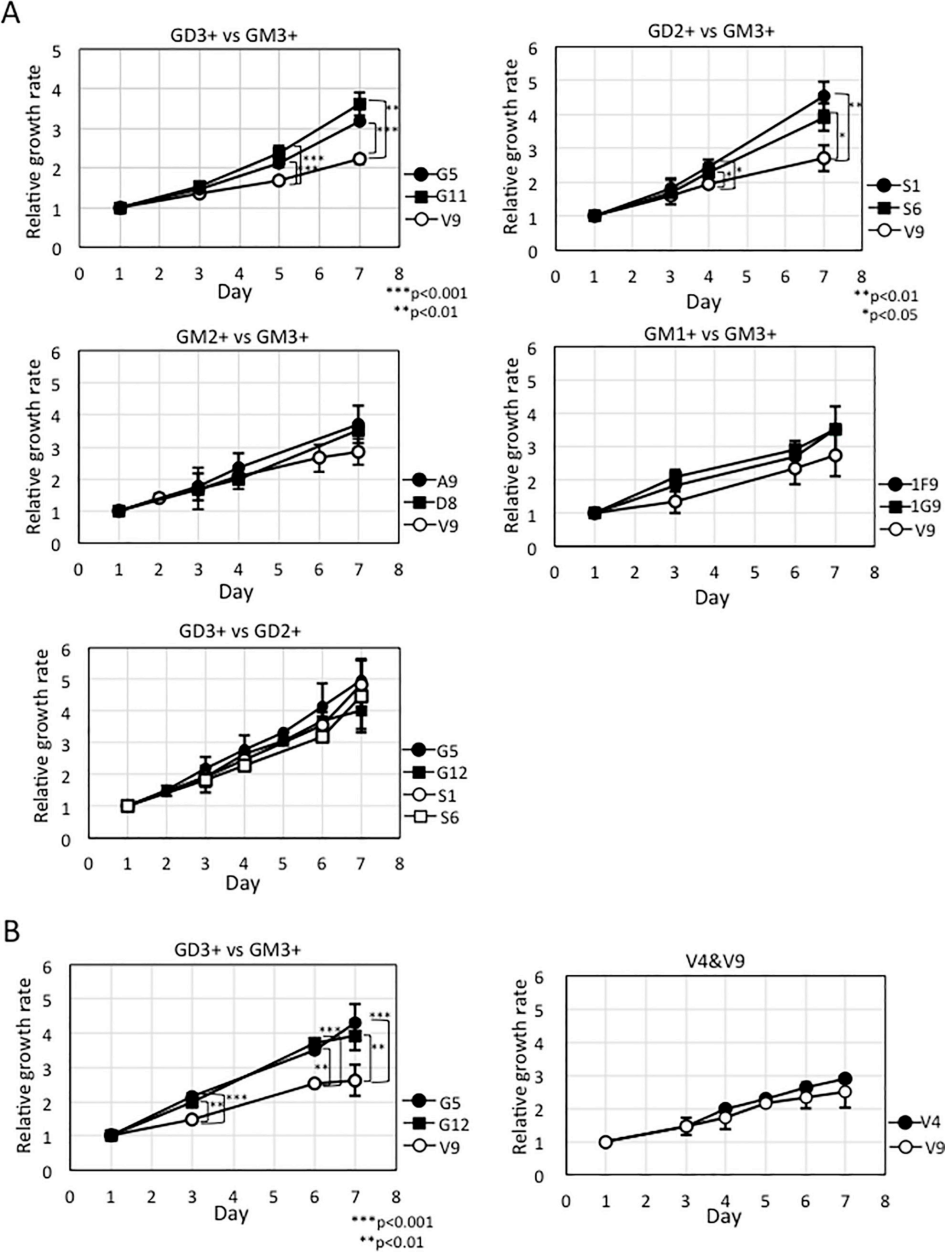
Cell growth activities of GD3+ cells and GD2+ cells increased. (A) Cell growth of various ganglioside-expressing cells and the control cells (V9) were analyzed by MTT assay (*upper*, *middle*). The cell growth of GD3+ cells and GD2+ cells were also compared (*lower*). (B) G12 is also a GD3+ cells and the cell growth are same as G5 (*left*). Cell growth of the control cells V9 and V4 were analyzed by MTT assay, indicating that the proliferation of a control line, V4 is almost same as a control line, V9 (*right*). The average of cell growth of each transfectant was calculated using the value of MTT assay performed by triplicate or quaternary samples. Bars are standard deviations (SDs). Data were analyzed by two-tailed Student’s t test (*, *p* <0.05; **, *p* <0.01; ***, *p* <0.001).

Results of MTT assay were shown in Fig 2. Compared with a control cells, V9, GD3+ cells and GD2+ cells showed increased proliferation, while GM2+ cells and GM1+ cells showed no significant differences with the control (Fig 2A). The cell proliferation of GD3+ cells and GD2+ cells was also compared (Fig 2A, *lower*), and there were no differences. Since the proliferation of GD3+ cells (G12) is almost same as G5 (Fig 2B, *left*), G12 was used for comparison of cell proliferation with GD2+ cells. The proliferation is almost same between two control cells, V4 and V9 (Fig 2B, *right*).

### Differential invasion activities were observed among individual groups

**Fig 3:**
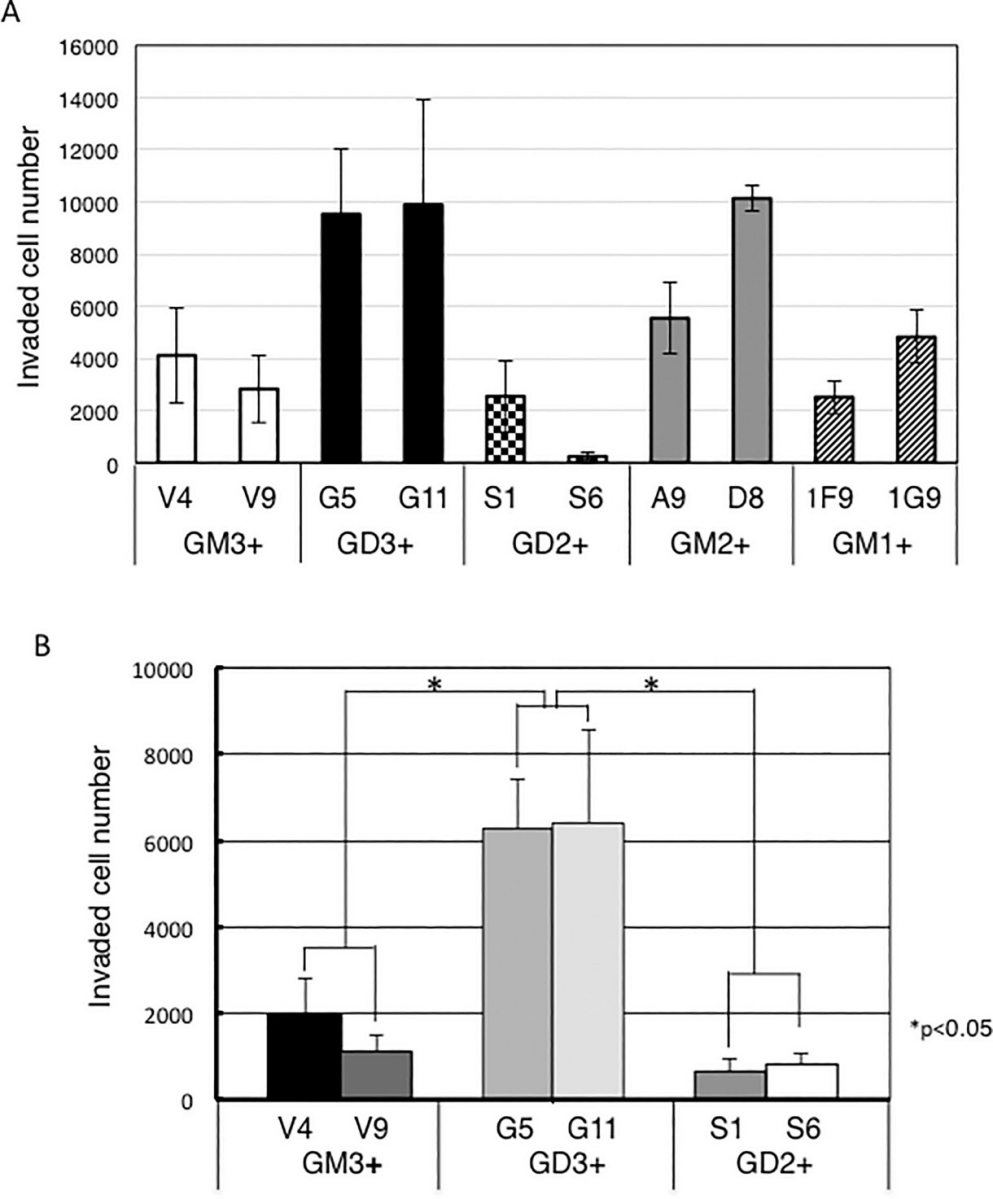
Invasion activities of GD3+ cells increased, while those of GD2+ cells rather reduced. Invasion activity of each transfectant was examined by invasion assay as described in Methods. (A) Invasion activities of GD3+, GD2+, GM2+, GM1+, and control cells were examined. (B) Further analysis of invasion activities with GM3+, GD3+ and GD2+ cells. Invasion activities of GD3+ cells significantly increased, while those of GD2+ cells were low compared with GD3+ cells. Mean values were presented. Bars are SDs (n = 3).

Results of invasion assay were shown as invaded cell number in Fig 3. GD3+ cells (G5, G11) showed higher number of cells than controls (V4, V9), while GD2+ cells (S1, S6) showed apparently lower invasion than GD3+ cells. As for GM2+ cells (A9, D8), they showed increased tendency compared with controls (Fig 3A). GD3+ cells and GD2+ cells as well as controls were examined again, since results of these cells were unstable with large SD. GD2+ cells showed significantly lower invasion than GD3+ cells, and reduced tendency compared with controls (Fig 3B). It seemed surprising that GD3+ cells and GD2+ cells showed different behaviors. We performed knockdown of *hB4GALNT1* gene in GD2+ cells to confirm decreased invasion activities of GD2+ cells (S1 Fig). Three of siRNAs (siRNA-6038, 6039 and 6041) of *hB4GALNT1* gene (S1A Fig) and a negative control siRNA were transfected into GD2+ cells. Expression levels of *hB4GALNT1* gene in the GD2+ cells transfected with individual siRNAs were examined at 3 days after the transfection, showing that siRNA-6039 was the most effective (S1B Fig).

GD3 expression level of cell surface on GD2+ cells S1 cells showed increased GD3 expression at one week after the transfection of siRNA-6039 (S1C Fig). GD3 expression on the GD2+ cells, S6 did not change at one week after the transfection with siRNA-6039 (data not shown). Then, siRNA-6039 was transfected once more into S6 cells. Consequently, GD3 expression level on the cell surface of S6 cells increased (S1C Fig). On the other hand, GD2 expression on S1 and S6 cells did not reduce after the transfection of siRNA-6039, probably because gangliosides stay on the cell surface for a long time as we previously reported [39]. The GD3-expressing GD2+ cells, at least S6 showed increased invasion activities and S1 showed a tendency to increase compared with cells transfected with control siRNA (S1D Fig). These results suggested that invasion activity was regulated by ganglioside profiles.

### GD3+ cells and GD2+ cells increased cell index in RT-CES with distinct intensity and kinetics

**Fig 4:**
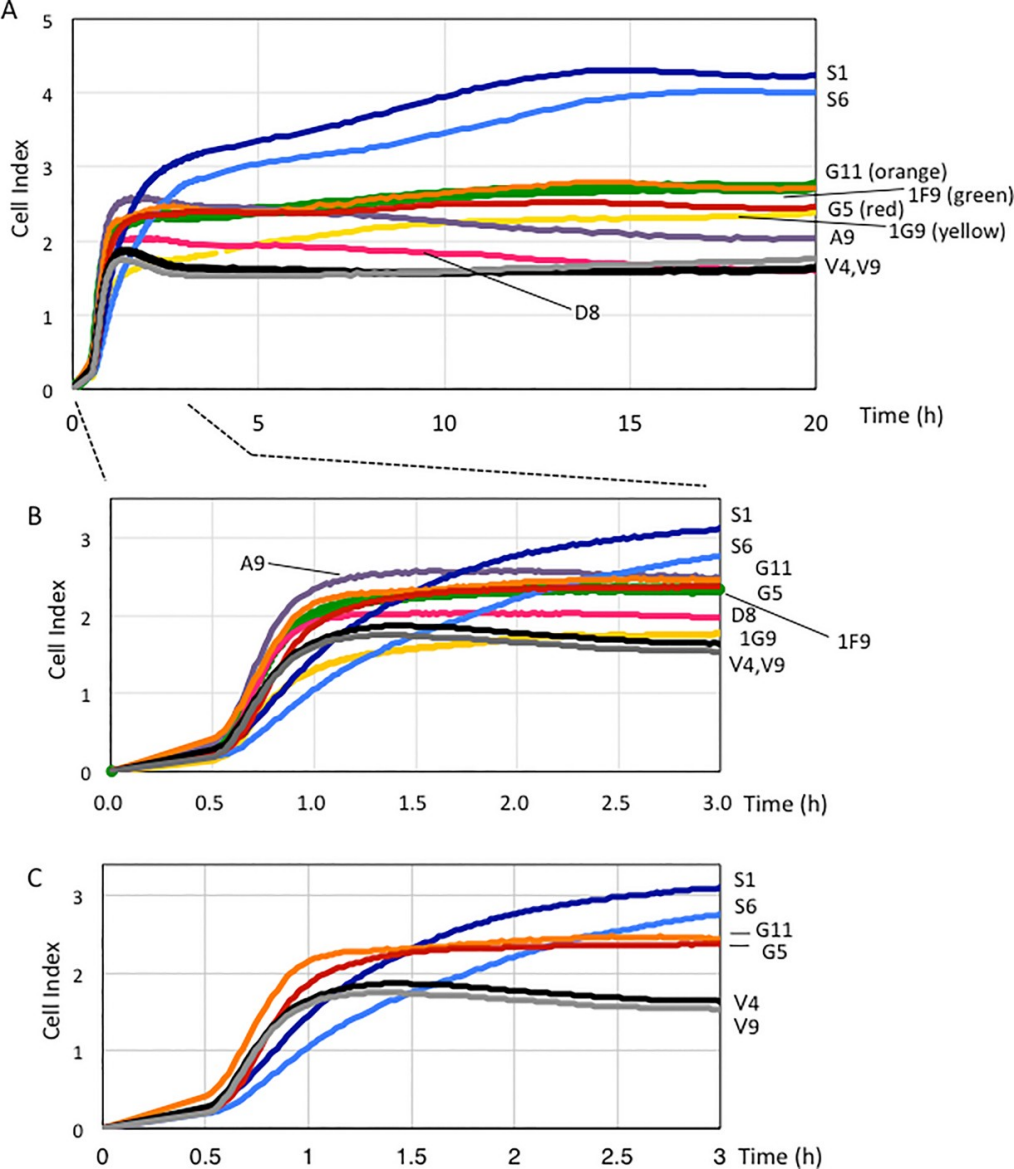
Cell adhesion of GD3+, GM1+ and GD2+ cells increased and GD2+ cells showed strongest adhesion/expansion at the later phase. (A) Intensities of adhesion and spreading of various ganglioside-expressing teransfectant cells and control cells (V4, V9) were analyzed by real-time cell electronic sensing (RT-CES). Intensity of cell adhesion and spreading was indicated as cell index (average of 3 or 4 samples) based on the electric resistance. Cell indices were recorded every 1 minute after adding cells into wells from 20 min to 3 h, and every 10 minutes from 3 h to 20 h. (B, C) The enlarged view of intensities of adhesion and spreading of various ganglioside-expressing teransfectant cells and control cells (B) and GD2+, GD3+ and control cells (C) during initial 3 h after starting of RT-CES.

RT-CES was performed to examine cell adhesion and subsequent expansion. Among 3 extracellular matrices, no essential differences were found in the adhesion patterns of GD2+, GD3+ and GM3+ control cells to CL-1 (S2 Fig). The cell adhesion and expansion of GD2+ cells to CL-IV and fibronectin also stronger than GD3+ and controla cells. Therefore, we performed RT-CES using CL-1 with all transfectant cells as shown in Fig 4. Five groups expressing different major gangliosides (GM3, GM2, GM1, GD3 or GD2) showed different patterns during long observation until 20 h (Fig 4A). In particular, GD2+ cells showed strongest adhesion/expansion among 5 groups. GD3+ cells and GM1+ cells also showed stronger adhesion activity than control cells. GM2+ cells also showed stronger adhesion than controls during first 12 h, but not so much at the later phase. The results of individual groups were showed in S3 Fig.

However, if we focus on the early phase, a few hours from the starting point, interesting fine differences among GD3+, GD2+ and GM3+ control cells could be seen as shown in Fig 4B and 4C in which cell indices of GD3+, GD2+ and GM3+ control cells were selected from Fig 4B to compare clearly. GD3+ cells quickly adhered to CL-1, and maintained the high adhesion levels, while GD2+ cells showed gradual increase of the cell index, exceeding the other groups and reaching the plateau at about 15 h after the starting point. These facts suggested that GD2+ cells slowly attached the palates, and gradually and intensively extended.

In the detachment experiment, GD2+ cells showed higher resistance to the treatment with 0.5 mM EDTA than GD3+ cells and GM3+ control cells as shown in S4 Fig.

### GD2+ cells showed reduced cell migration velocity in time-lapse imaging

**Fig 5:**
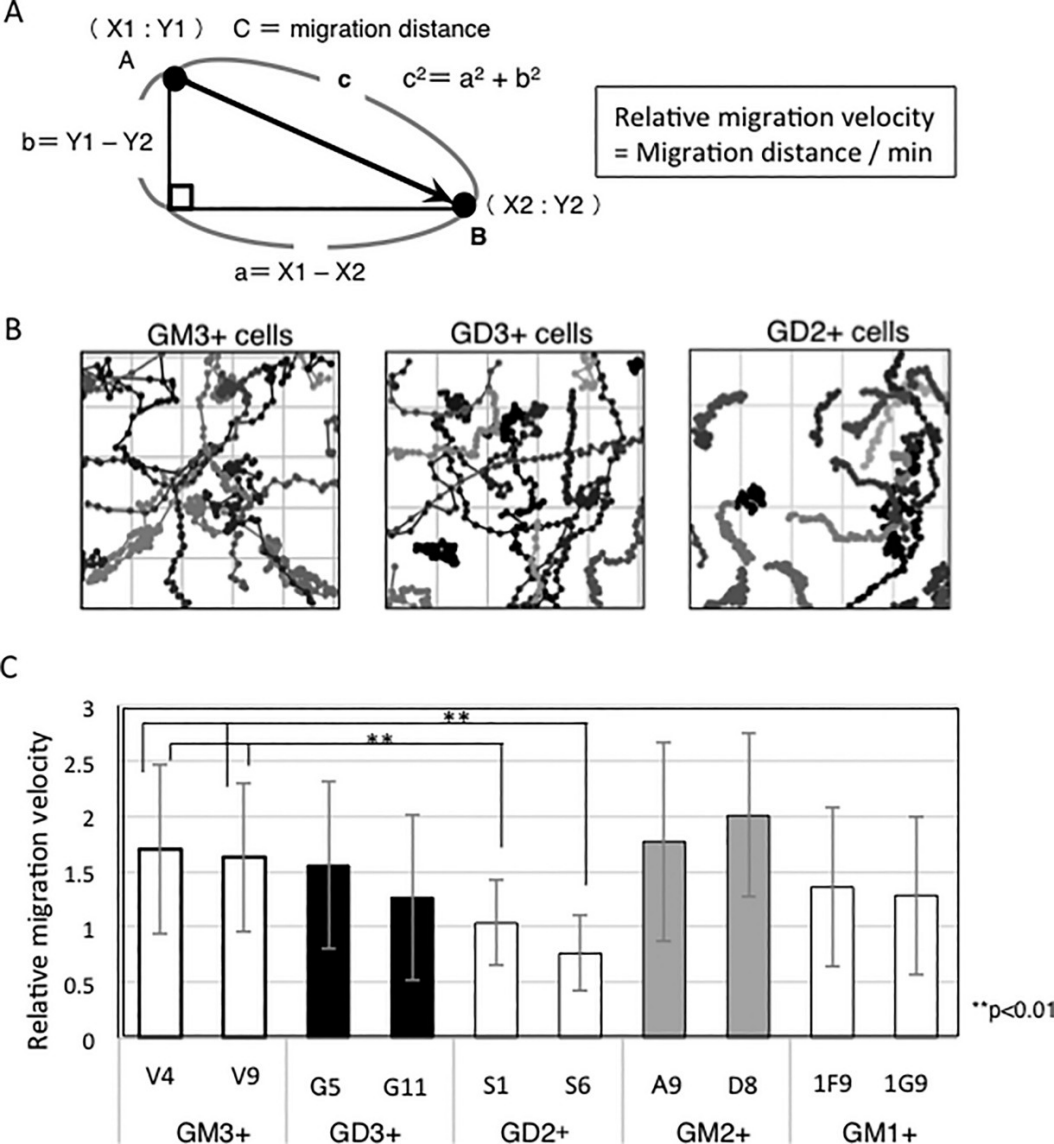
Cell migration of GD2+ cells was slower than other transfectant cells in time-lapse imaging. (A) Calculation of relative migration velocity of cells. Cell migration was observed by a confocal laser microscope. X- and Y-position of cells at each time was recorded and migration distance was calculated. Relative migration velocity of a cell was calculated by dividing total migration distance by whole recording time (min). (B) Examples of plots of migrating cells recorded for 20 h. (C) Relative migration velocities of various ganglioside-expressing cells. The average of relative migration velocity of each transfectant was calculated using migration velocities of individual cells. Numbers of cells examined: V4 (n = 33), V9 (n = 36), G5 (n = 36), G11 (n = 47), S1 (n = 36), S6 (n = 47), A9 (n = 36), D8 (n = 31), 1F9 (n = 40), 1G9 (n = 44). Bars are SDs.

Cell migration was observed by confocal laser microscopy, and their migration distances were calculated by measuring moving traces as shown in Fig 5A and 5B. Although the migration velocity of cells was quite dispersed even in a same group, GD2+ cells showed generally slower patterns than the other groups (Fig 5C).

### Localization of gangliosides as analyzed by immunocytostaining

**Fig 6:**
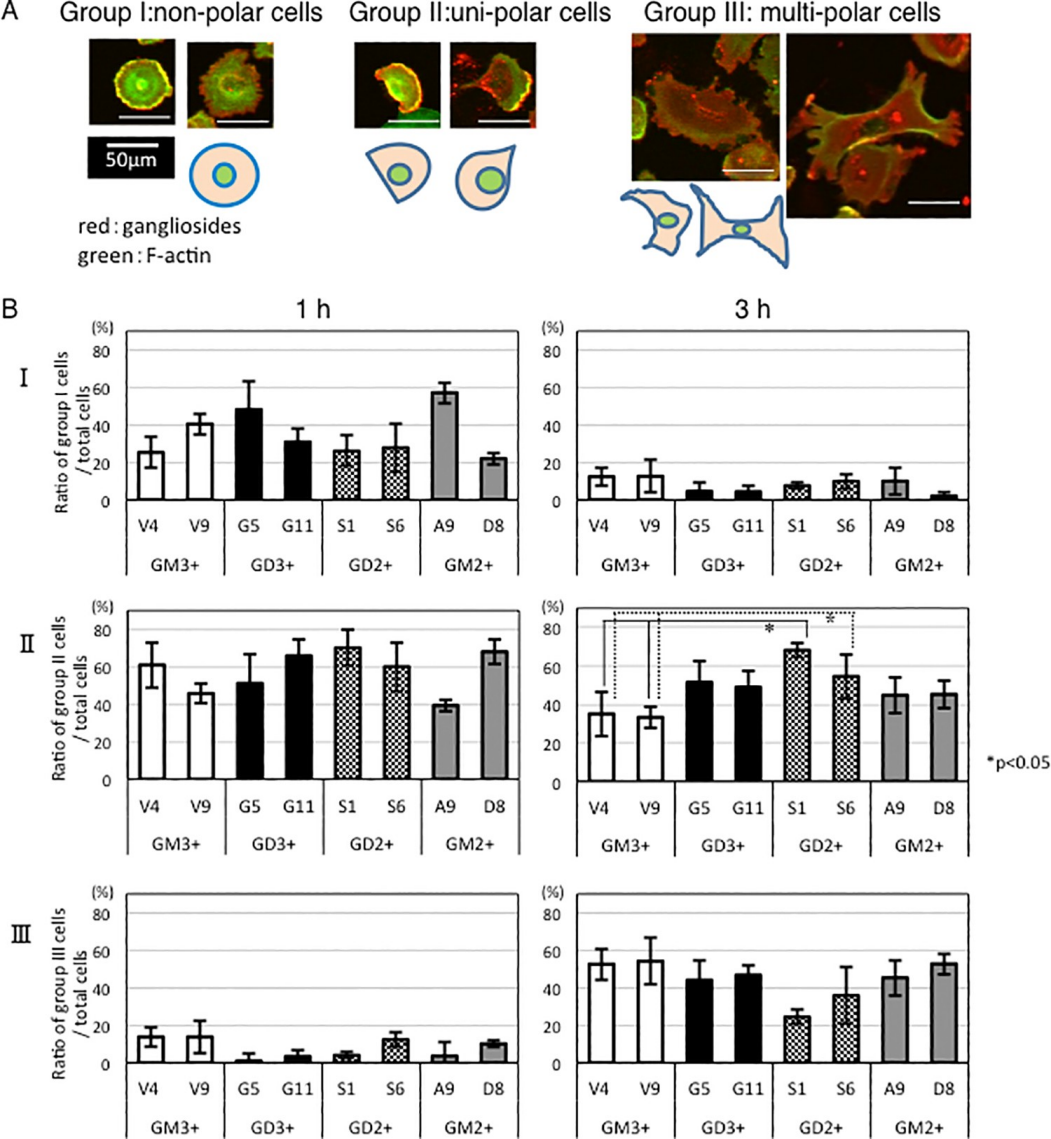
Morphological classification of transfectant cells during cell adhesion to CL-I. (A) Cells were classified into types, I, II, and III. I represented non-polar cells. II represented uni-polar cells, and III were multi-polar cells as shown. Cells were fixed at 1 h and 3 h after cell adhesion to CL-I-coated plates and double immunostaining of F-actin (green) and gangliosides (red) was performed. (B) Cell morphology was observed by a confocal laser microscope and individual transfectants were classified into three groups. Results at 1 h and 3 h were demonstrated. GD2+ cells tended to keep uni-polar pattern (II) at 3 h, while other cells tended to change to multi-polar group (III). Numbers of cells examined at 1 h: V4 (n = 169), V9 (n = 175), G5 (n = 156), G11 (n = 130), S1 (n = 212), S6 (n = 189), A9 (n = 191), D8 (n = 139). Numbers of cells examined at 3h: V4 (n = 189), V9 (n = 166), G5 (n = 355), G11 (n = 170), S1 (n = 212), S6 (n = 230), A9 (n = 166), D8 (n = 154). Bars are SDs.

Cells were classified into three groups according to cell shapes (Fig 6). Group I is those having no polarity frequently detected at early phase (1 h) during cell adhesion to CL-I-coated plates. Group II is those containing polar cells that were migrating toward single direction. Multi-polar cells that appeared to migrate toward multiple directions were classified as group III (Fig 6A). At the early phase (~1 h), ratio of Group II (polar cells) was generally high in all kinds of cells as well as that of Group I. At 3 h after cell plating and adhesion, the ratio of Group I generally reduced, and that of Group III increased. Although ratio of Group II was still high in all cells at 3 h, GD2+ cells showed the highest ratio compared with group I and III, and also being significantly higher than GM3+ control cells. The ratio of group III (multi-polar cells) was increasing in GD3+, GM2+ cells, and GM3+ control cells, while it was not so much in GD2+ cells (Fig 6B).

**Fig 7:**
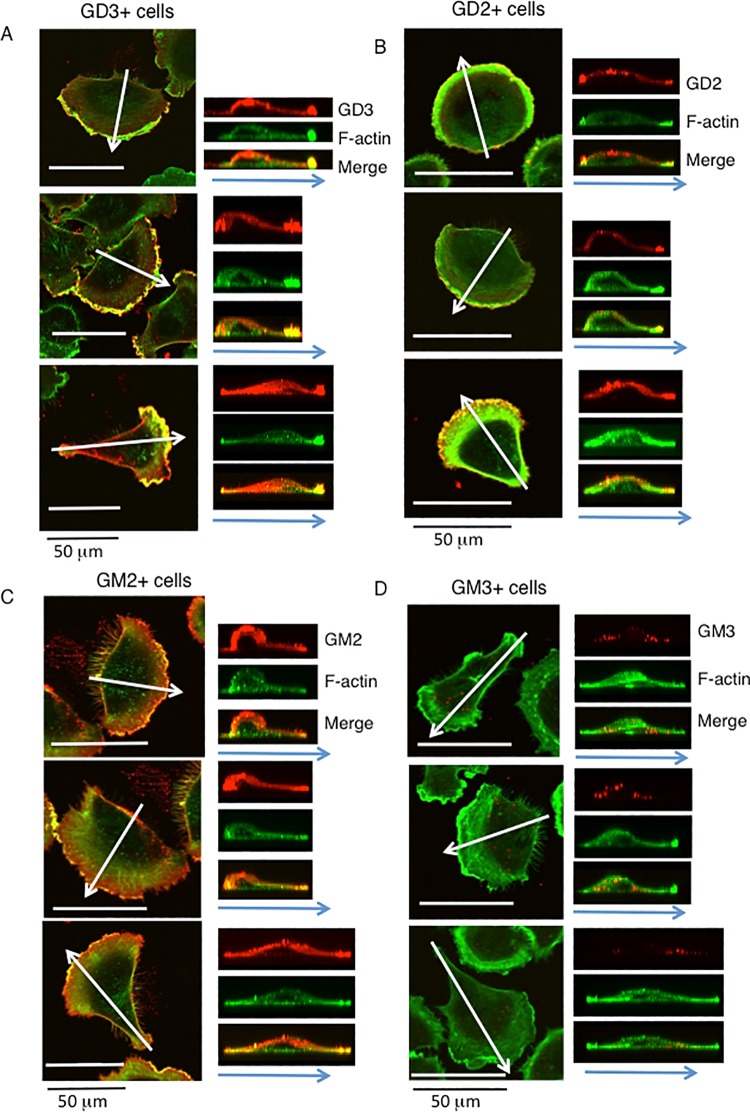
Double immunostaining of F-actin and gangliosides of GD3+ and GD2+ cells showed clear co-localization at the leading edges of cells. Cells were fixed at 1 h after cell adhesion to CL-I-coated plates. Double immunostaining of F-actin (green) and gangliosides (red) was performed as described in Methods, and cells were observed by a confocal laser microscope. Results of GD3+ cells (A), GD2+ cells (B), GM2+ cells (C) and GM3+ control cells (D) classified into type II in Fig 6 were shown. The images of the z-axis were shown at the right side of individual images. White arrows indicate the site used for z-axis imaging. Blue arrows indicate the direction of cell migration.

Cells were fixed, and double immunostaining of F-actin and gangliosides was performed by Fluor 488-phalloidin and antibodies reactive with individual gangliosides, respectively (Fig 7). F-actin and GD3 or GD2 were co-localized at the leading edges of cells. F-actin and GM2 were also co-localized at the leading edge but co-localizing patterns were weaker than that of GD3 or GD2 (Fig 7). It was difficult to detect GM3 with anti-GM3 mAb at the leading edges, while leading edges were stained by F-actin in GM3+ control cells.

### Phosphorylation of signal proteins upon FCS stimulation was enhanced in GD3+ cells but not in GD2+ cells, however phosphorylation of Akt was enhanced in both cells

GD3+ cells and GD2+ cells showed increased cell growth, while their invasion activity was different. Namely, GD3+ cells showed enhanced invasion, while GD2+ cells did not show enhanced invasion activity, and showed low migration velocity compared with GM3+ control cells. In stead, GD2+ cells showed clearly increased adhesion and expansion to ECM. Since we previously reported that phospholylations of p130Cas, paxillin and Akt were enhanced in GD3+ cells compared with GM3+ control cells [29,40,41], we tried to clarify mechanisms for these differences in GD3+ and GD2+ cell phenotypes. Western immunoblotting with an anti-phosphotyrosine antibody (PY20) was performed and the band at about 60 kDa in GD2+ cells, which may be paxillin, was weaker than that in GD3+ cells (S5 Fig). Then, western immunoblotting with site specific anti-phosphotyrosine-p130Cas antibodies and anti-phospho-paxillin antibodies, and also anti-phospho-Akt antibodies were performed using cell lysates prepared from FCS treated cells.

**Fig 8:**
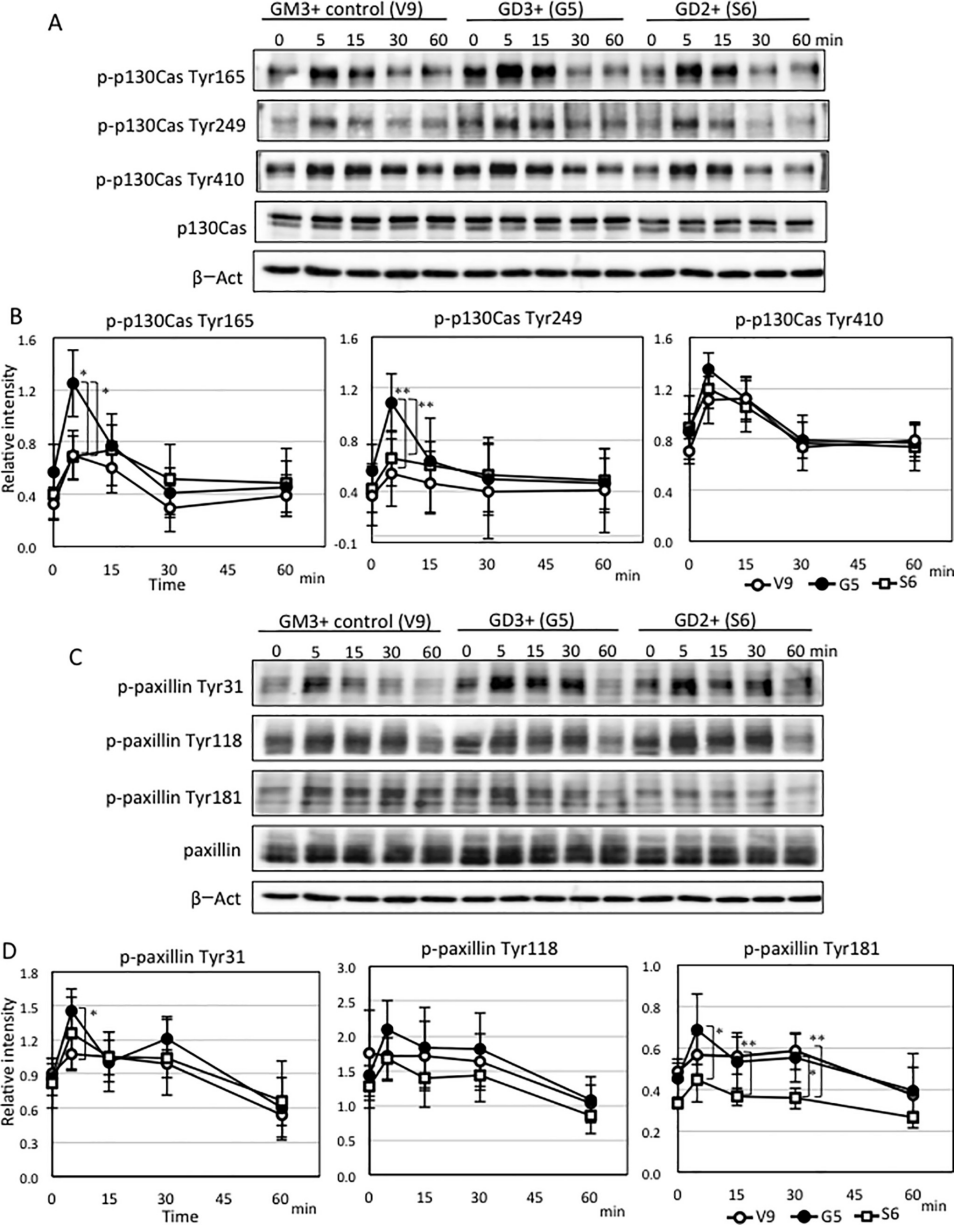
Changes in phosphorylation levels of proteins after FCS treatment in GD3+ cells, GD2+ cells and control cells. (A) To analyze proteins involved in the cellular phenotypes of GD3+ cells and GD2+ cells, western immunoblotting with anti-phospho-p130Cas antibodies (p-p130Cas Tyr165, Tyr249, Tyr410) were performed using cell lysates prepared after FCS treatment (0, 5, 15, 30 and 60 min). Cells were plated in dishes and serum-starved for 20 h before FCS treatment. (C) Western immunoblotting with anti-phospho-paxillin antibodies (p-paxillin Tyr31, Tyr118, Tyr181) were performed using cell lysates prepared after FCS treatment. (E) Western immunoblotting with anti-phospho-Akt antibodies (p-Akt Ser473, Thr308) were performed using cell lysates prepared after FCS treatment. (B, D, F) Band intensity was quantified by Amersham Imager 680, and relative intensity of bands was plotted after correction with that of ß-actin. These experiments were repeated three or four times. Bars indicate SDs. Data were analyzed by two-tailed Student’s t test (*, *p* <0.05; **, *p* <0.01).

As shown in Fig 8A and 8B, p130Cas showed similar tyrosine phosphorylation patterns as we previously reported for GD3+ cells and GM3+ control cells [29,40]. p130Cas in GD2+ cells was not activated more than control GM3+ control cells. As for paxillin, GD3+ cells showed a tendency to increase the phosphorylation and Tyr31 was activated more strongly in GD3+ cells than in control cells as reported [29,40]. Interestingly, GD2+ cells showed lower phosphorylation levels of Tyr181 even than GM3+ control cells (Fig 8C and 8D). Phosphorylation levels of Akt in GD3+ and GD2+ cells were enhanced compared with those in GM3+ control cells. In particular, phosphorylation of Thr308 at 15 min and 60 min in GD2+ cells was significantly enhanced compared with GD3+ cells (Fig 8E and 8F). These results seemed to be basis for the differential effects of individual gangliosides.

**Fig 9:**
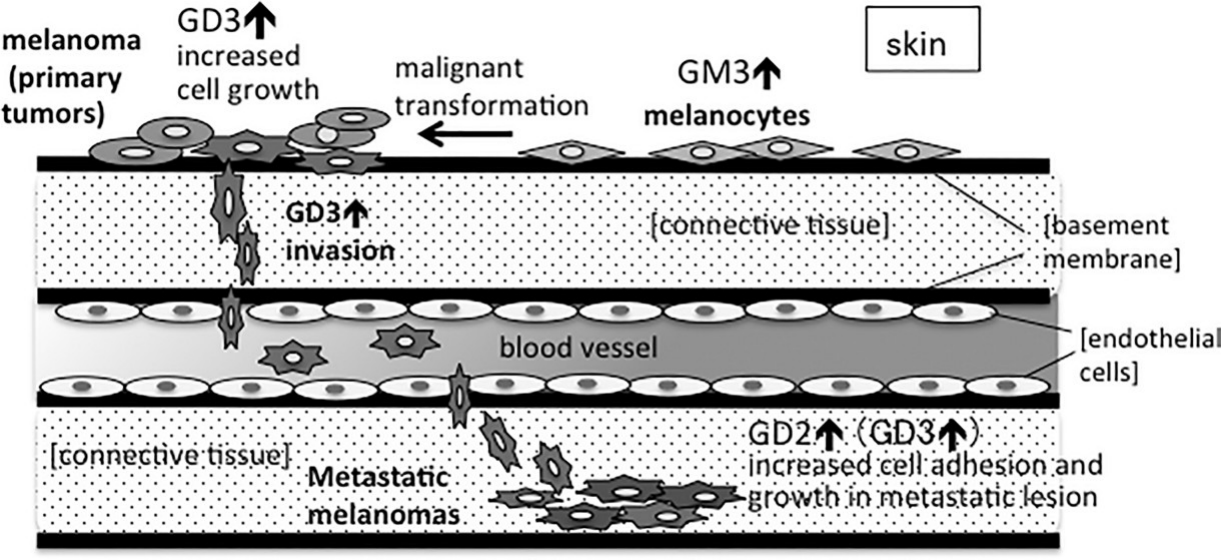
Differential roles of gangliosides in the individual processes of progression and metastasis of melanomas. Results obtained in this study and these in past articles from our group and other groups were combined in this schema.

Supposed roles of individual gangliosides based on the results of this study as well as many past articles [29,40,42–46] were summarized in Fig 9. GD3 is expressed along with development of malignant melanomas from melanocytes, inducing malignant cancer properties. GD3 is also persistently expressed during tumor expansion and invasion into surrounding tissues, leading to metastasis. On the other hand, GD2 is mainly expressed at the later phase, conferring solid adhesion and fixation as well as rapid cell growth at the metastasized sites.

## Discussion

There have been a number of reports on the roles of gangliosides in the phenotypes and behaviors of normal cells [47] and malignant cells [48]. In particular, roles of gangliosides in cancer cells have been paid a lot of attention by many researchers, resulting in the reports on interesting aspects of their functions in individual cell lineages. However, no comprehensive analysis of various gangliosides expressed on the cell surface based on same cellular backgrounds has been performed. In this study, we have established multiple transfectant cells expressing various gangliosides as main components based on genetic modification of expression profiles of gangliosides by using a melanoma cell line, SK-MEL-28 N1 [34]. Comparative analysis of these transfectant cells revealed that individual groups of transfectants with characteristic ganglioside expression exhibited distinct phenotypes and behaviors in cell growth, invasion, migration and adhesion/spreading as analyzed by RT-CES.

Although it has been generally believed that sialic acids attached on the membrane molecules strongly affect phenotypes and behaviors of cells [49,50]. Number of sialylated sites on glycoproteins and glycolipids and structures of sialylated carbohydrates on them are actually critical for the interaction between those sugar chains and their ligands. For gangliosides, we previously reported that monosialyl gangliosides and disialyl gangliosides induce opposite properties of cells and contrastive regulation of intracellular signaling [22,28–32,51–54]. Even among disialyl gangliosides, tandem-repeat disialyl gangliosides such as GD3 and GD2 and branched-type disialyl gangliosides like disialyl Lewis a and disialyl galactosylgloboside showed different effects on cancer cells and/or normal cells [55–57].

Tandem-repeated sialyl structures should be important for increased cell growth, invasion, mobility and probably metastasis, while effects of expression of GD3/GD2 might be slightly different depending on cell lineages [36,46,58,59]. Not only carbohydrate moiety of glycosphingolipids on the cell surface, but also cytoplasmic signaling molecules in individual cells, and their combination seem to lead cells to particular directions of phenotypes and behaviors.

Previously, it was reported that GD2 was expressed in melanomas at late, vertical invasion stage, and probably functions for the induction of cancer metastasis [44,45]. GD2 was also reported to be critical for the survival of SCLC cells by increasing cell adhesion to ECM [28], or to be important for the activated growth signals in breast cancers [60]. All these results suggested that GD2 and GD3 play positive roles for the enhancement of cancer cell growth, adhesion, and also cell migration in a similar manner. The results obtained in this study indicated that GD3 and GD2 actually exert enhancement of malignant properties by distinct directions, while they frequently behave almost similarly. As shown in Fig 2, GD3 and GD2 enhanced cell growth of melanoma cells. But in cell invasion, migration, and adhesion/spreading, they showed quite different effects on the individual transfectant cells. Although it has been expected that GD2 expression enhances cell detachment from primary sites, and cell migration, leading to remote metastasis, the transfectant cells almost exclusively expressing GD2 exhibited extremely increased cell adhesion/spreading, reduced invasion and migration. Therefore, it was suggested that GD2 plays roles in the attachment and fixation on the metastatic sites by keeping increased capability of cell growth as shown in Fig 9. GD2 is frequently expressed together with GD3 in some melanomas, gliomas, SCLCs and osteosarcomas [28–30,59]. Therefore, simultaneous expression of these disialyl gangliosides has hampered correct understanding of roles of individual structures. As shown in this study, the transfectant cells expressing single major gangliosides except GM1-expressing ones enabled us to clarify specific roles for each ganglioside structure.

It was reported that signal transduction by GD3 is involved in growth cone morphology via tyrosine phosphorylation of paxillin and F-actin assembly in cerebellar granule cells [61]. Gangliosides were also reported to modulate dendritic morphogenesis via CaM-KII and cdc42-mediated actin organization [62]. A critical role of sphingolipid composition in regulating the lateral mobility of integrins and their ability to dynamically increase receptor density was also reported [63]. Thus, close localization of gangliosides and F-actin in lamellipodia strongly suggests that gangliosides regulate cell mobility, adhesion and invasiveness by differentially interact with various membrane molecules and/or cytoplasmic molecules, leading to the regulation of cytoskeletons in the vicinity of cell membrane. In particular, precise roles of gangliosides in invadosomes (lamellipodia) and in uropodia are very intriguing issues and remain to be urgently analyzed. Although majority of gangliosides should be expressed on the cell surface, some gangliosides were found in cytoplasm, probably playing unknown roles [64].

In the analysis of signal activation, p130Cas did not show increased activation in GD2+ cells in contrast with GD3+ cells that we reported previously [29]. Although p130Cas is considered to be a key signaling node for interactions with multiple proteins to regulate normal and pathological cell functions [65], p130Cas might not be highly activated by serum stimulation in GD2+ cells (Fig 8A and 8B). Paxillin is a multi-domain protein localizing primarily to sites of cell adhesion to the extracellular matrix [66]. Paxillin binds to many proteins involved in effecting changes in the organization of the actin cytoskeleton, which is needed for cell motility events including cancer metastasis [66]. In particular, paxillin phosphorylation is considered to regulate cell spreading and migration [67]. Mutations of Tyr31 and Tyr118 in some cell lines inhibited cell migration, while transfection of wild type paxillin into another cell lines inhibited cell migration [68]. Thus, the paxillin Tyr31/Tyr118 mutation either promoted or inhibited cell migration depending on cell types [67]. Actually, phosphorylation of paxillin Tyr31 and Tyr118 regulated actin-dependent polalization and motility of pre-B cell line [69]. Therefore, increased phosphorylation of paxillin Tyr31 in addition to increased phosphorylation of p130Cas in GD3+ cells [67] may play roles in the increased invasion (Fig 3) as reported in skeletal muscle satellite cells [70]. Reduced Tyr181 phosphorylation in addition to low level of p130Cas phosphorylation in GD2+ cells might be involved in the lowered invasion/migration activity, while it is not clear at this moment. Since GD2 was reported to be a cancer stem cell marker of breast cancer [71], it may be implicated in rather static nature during cancer development.

As for increased Akt phosphorylation, both pSer473 and pThr308 showed increase in GD3+ and GD2+ cells, particularly pThr308 showed marked increase in GD2+ cells even when compared with GD3+ cells. Akt has been known to have greatly expanded functional repertoire [72]. A number of studies have revealed various physiological roles of Akt network, and roles in diverse pathological settings including cancers [72]. Activation of Akt is necessary for growth, proliferation, metastasis [73], and contribute to chemo- and radioresistance [74]. The facts that Akt activation is deeply linked with metastasis and therapy resistance might correspond with supposed functions of GD2 in advanced melanomas [44]. Strong activation of Akt may contribute in the vertical growth and metastasis [73] as well as increased cell growth of GD2+ cells (Fig 2). Akt might also contribute in the increased cell adhesion and expansion detected in GD2+ cells at the later phase of RT-CES (Fig 4) as we already reported its roles in the adhesion to collagens of GD3 [40], and in the increased HGF/Met signals by GD3 [41]. The mechanisms for the markedly increased cell adhesion and expansion detected in GD2+ cells at the later phase of RT-CES remain to be investigated in the future.

GM1 has been considered as a representative GEM/raft marker [75]. However, we have reported its regulatory function in lipid rafts [76] as a main functional resident of the microdomains similarly to caveolin-1 [32,52,77–79]. In this study, expression of GM1 resulted in the reduced cell growth and invasion compared with GD3/GD2-expressing cells. These phenotypes of GM1+ cells might reflect those of the normal counter part of melanomas, melanocytes, in which GM1 synthase is expressed at higher levels than in melanomas [80].

As we have recently reported, glycosphingolipids are involved in the regulation of cell signaling transduced via GEM/rafts based on the interaction with and modulation of various membrane molecules [36,81,82]. They often form molecular complex on the cell surface including intracellular molecules such as Src family kinases [36]. The mechanisms for the differential effects of individual gangliosides on the cell phenotypes as shown in this study might depend on what kinds of molecules are involved in the complex formation with individual ganglioside species. Identification and investigation of profiles of membrane molecules associating with individual gangliosides remain to be analyzed.

## Supporting information

S1 Fig Invasion activities of GD2+ cells increased by knockdown of *hB4GALNT1* gene. (A) Sequences of siRNA for *hB4GALNT1* genes (G, C, U and A, RNA base; T; DNA base). MISSION siRNA Universal Negative Control (Sigma-Aldrich) was used as a control of siRNA. (B) Expression levels of *hB4GALNT1* gene in siRNA-transfected cells (*upper*: GD2+ cells, S1; *lower*: GD2+ cells, S6) were evaluated by real-time RT-PCR, and results were presented after correction with *hGAPDH* gene. Every sample was measured in duplicate, and data were shown as mean ± SD. (C) Flow cytometry of ganglioside expression on siRNA-transfected cells (*upper*, S1; *lower*, S6. red line, siRNA-6039; blue line, siRNA-control) GD3 and GD2 were detected with mAb R24 and mAb 220–51, respectively. (D) Invasion activities of siRNA-6039 or siRNA-control-transfected GD2+ expressing cell (*Left*, S1; *right*, S6) were examined. Data were shown as mean ± SD (*n* = 2 for S1 melanoma, *n* = 3 for S6). Data were analyzed by two-tailed Student’s t test (*, *p*<0.05). Increase in GD3-positive population in flow cytometry corresponded with increased invasion activity.
(TIF)

S2 Fig Cell adhesion to various ECM measured by RT-CES. Cell adhesion was compared with various ECM using RT-CES plates pre-coated with various ECM. One x 10^4^ cells were plated in pre-coated plates, and adhesion activity was measured for 0~12 h. Used ECM were collagen type I (CL-I), collagen type IV (CL-IV) and fibronectin (FN). Consequently, adhesion activity was as GD2+ > GD3+ > VC for all ECM (CL-I, CL-IV and FN).
(TIF)

S3 Fig Cell adhesion increased in GD3+, GM1+ and GD2+ cells. Intensities of adhesion and spreading of individual ganglioside-expressing cells and control cells (V4, V9) as measured by RT-CES were shown. Intensity of cell adhesion and spreading was indicated as cell index based on the electric resistance. Cell indices were recorded after adding cells into wells every 1 minute from 20 min to 3 h and every 10 minutes from 3 h to 20 h. Bars on individual marks indicate SDs (n = 3 or 4). Data at 3 h, 5 h, 10 h, 15 h, and 20 h were analyzed by two-tailed Student’s t test. All *p*-values between GD3+ and control cells, and between GD2+ cells and controls were *p* <0.05. The *p*-values between GM1+ cells and control cells after 5 h were *p* <0.05.
(TIF)

S4 Fig Resistance of GD2+ cells to detachment with EDTA treatment. In usual experiments, GD2+ cells were highly resistant to detachment with EDTA treatment. Then, we examined wheather GD2+ cells are resistant to EDTA. At 3, 5, 10 and 15 min after EDTA treatment, detachment of cells were analyzed by morphology (A) and by counting detached cell number (B). GD2+ cells were resistant to detachment. These data suggested that GD2+ cells strongly adhere to plates in a cadherin-independent manner.
(TIF)

S5 Fig Tyrosine phosphorylation of proteins after FCS treatment in GD3+ cells, GD2+ cells and GM3+ control cells. To analyze proteins involved in the cellular phenotypes of GD3+ and GD2+ cells, western immunoblotting with an anti-phosphotyrosine antibody (PY20) was performed using cell lysates prepared after FCS treatment. Cells were plated in dishes and serum-starved for 20 h before the treatment with FCS.
(TIF)
