# Correction: Differential roles of gangliosides in malignant properties of melanomas

**DOI:** 10.1371/journal.pone.0222220

**Published:** 2019-09-03

**Authors:** The PLOS ONE Staff

In Fig 8, panels E and F do not appear. The publisher apologizes for the error. Please see the complete, correct Fig 8 here.

**Fig 8:**
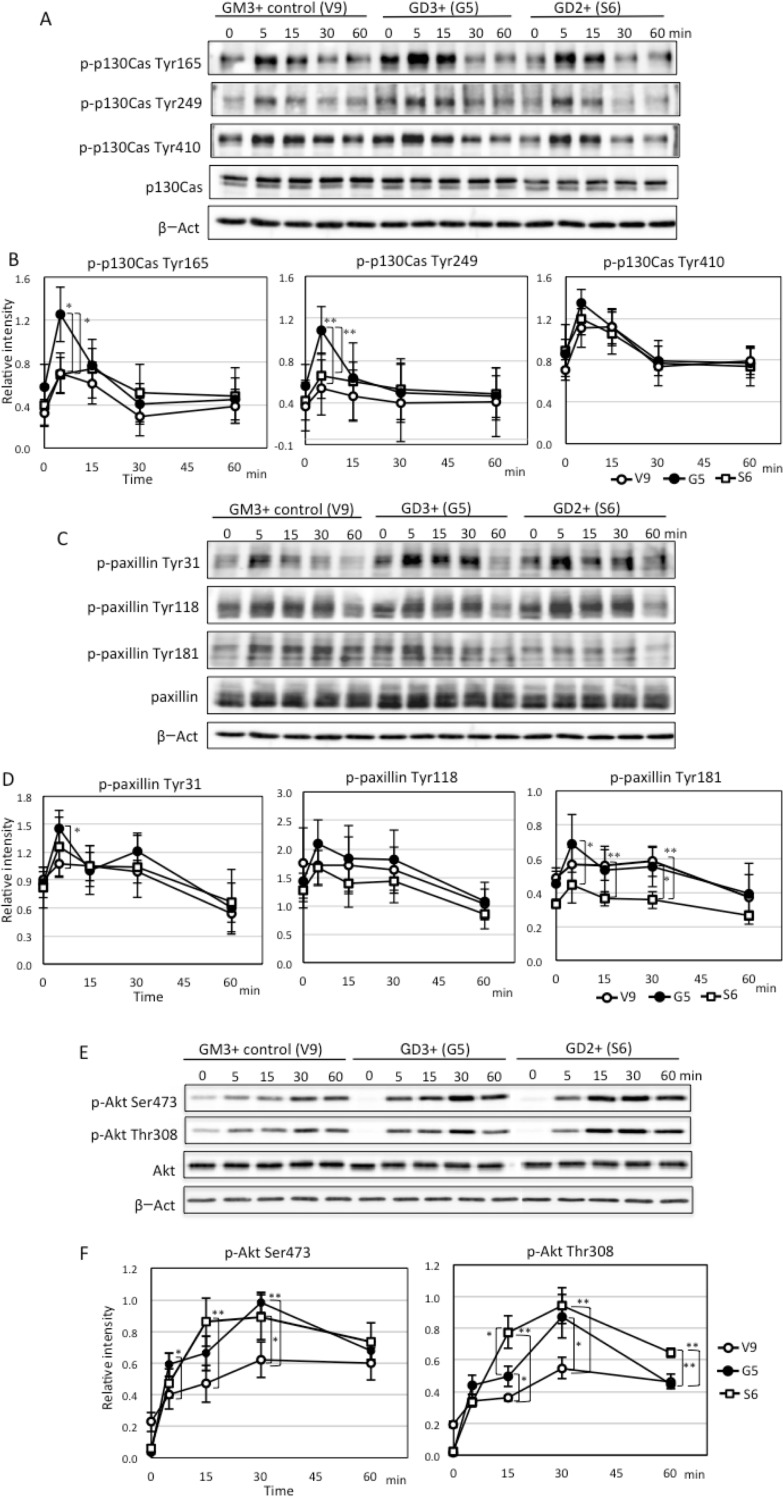
Changes in phosphorylation levels of proteins after FCS treatment in GD3+ cells, GD2+ cells and control cells. (A) To analyze proteins involved in the cellular phenotypes of GD3+ cells and GD2+ cells, western immunoblotting with anti-phospho-p130Cas antibodies (p-p130Cas Tyr165, Tyr249, Tyr410) were performed using cell lysates prepared after FCS treatment (0, 5, 15, 30 and 60 min). Cells were plated in dishes and serum-starved for 20 h before FCS treatment. (C) Western immunoblotting with anti-phospho-paxillin antibodies (p-paxillin Tyr31, Tyr118, Tyr181) were performed using cell lysates prepared after FCS treatment. (E) Western immunoblotting with anti-phospho-Akt antibodies (p-Akt Ser473, Thr308) were performed using cell lysates prepared after FCS treatment. (B, D, F) Band intensity was quantified by Amersham Imager 680, and relative intensity of bands was plotted after correction with that of ß-actin. These experiments were repeated three or four times. Bars indicate SDs. Data were analyzed by two-tailed Student’s t test (*, *p*<0.05; **, *p* <0.01).
