# Supplementary figures and images for: Differential roles of gangliosides in malignant properties of melanomas
Source: PLoS One. 2018 Nov 21;13(11):e0206881. doi: 10.1371/journal.pone.0206881 (PMC6248923; doi:10.1371/journal.pone.0206881)

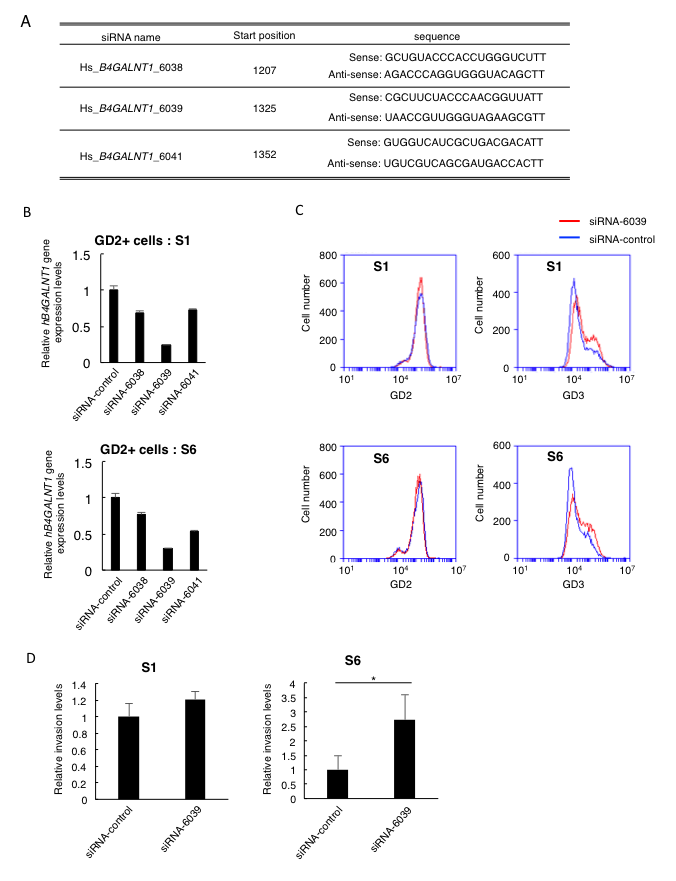

Supplement: S1 Fig — (A) Sequences of siRNA for hB4GALNT1 genes (G, C, U and A, RNA base; T; DNA base). MISSION siRNA Universal Negative Control (Sigma-Aldrich) was used as a control of siRNA. (B) Expression levels of hB4GALNT1 gene in siRNA-transfected cells (upper: GD2+ cells, S1; lower: GD2+ cells, S6) were evaluated by real-time RT-PCR, and results were presented after correction with hGAPDH gene. Every sample was measured in duplicate, and data were shown as mean ± SD. (C) Flow cytometry of ganglioside expression on siRNA-transfected cells (upper, S1; lower, S6. red line, siRNA-6039; blue line, siRNA-control) GD3 and GD2 were detected with mAb R24 and mAb 220–51, respectively. (D) Invasion activities of siRNA-6039 or siRNA-control-transfected GD2+ expressing cell (Left, S1; right, S6) were examined. Data were shown as mean ± SD (n = 2 for S1 melanoma, n = 3 for S6). Data were analyzed by two-tailed Student’s t test (*, p<0.05). Increase in GD3-positive population in flow cytometry corresponded with increased invasion activity. (TIF) [file pone.0206881.s001.tif]

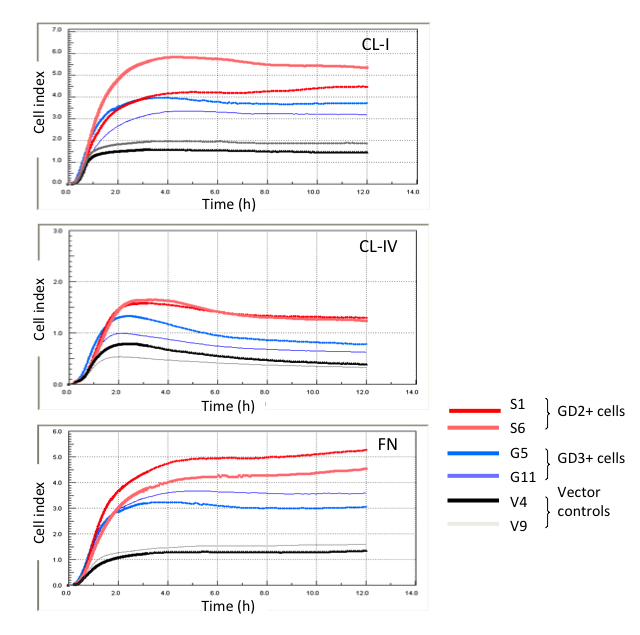

Supplement: S2 Fig — Cell adhesion was compared with various ECM using RT-CES plates pre-coated with various ECM. One x 104 cells were plated in pre-coated plates, and adhesion activity was measured for 0~12 h. Used ECM were collagen type I (CL-I), collagen type IV (CL-IV) and fibronectin (FN). Consequently, adhesion activity was as GD2+ > GD3+ > VC for all ECM (CL-I, CL-IV and FN). (TIF) [file pone.0206881.s002.tif]

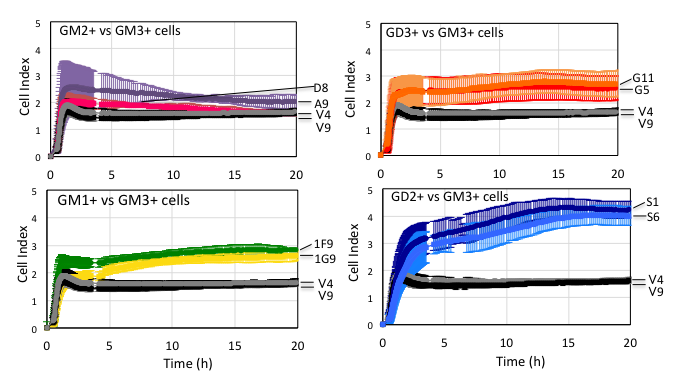

Supplement: S3 Fig — Intensities of adhesion and spreading of individual ganglioside-expressing cells and control cells (V4, V9) as measured by RT-CES were shown. Intensity of cell adhesion and spreading was indicated as cell index based on the electric resistance. Cell indices were recorded after adding cells into wells every 1 minute from 20 min to 3 h and every 10 minutes from 3 h to 20 h. Bars on individual marks indicate SDs (n = 3 or 4). Data at 3 h, 5 h, 10 h, 15 h, and 20 h were analyzed by two-tailed Student’s t test. All p-values between GD3+ and control cells, and between GD2+ cells and controls were p <0.05. The p-values between GM1+ cells and control cells after 5 h were p <0.05. (TIF) [file pone.0206881.s003.tif]

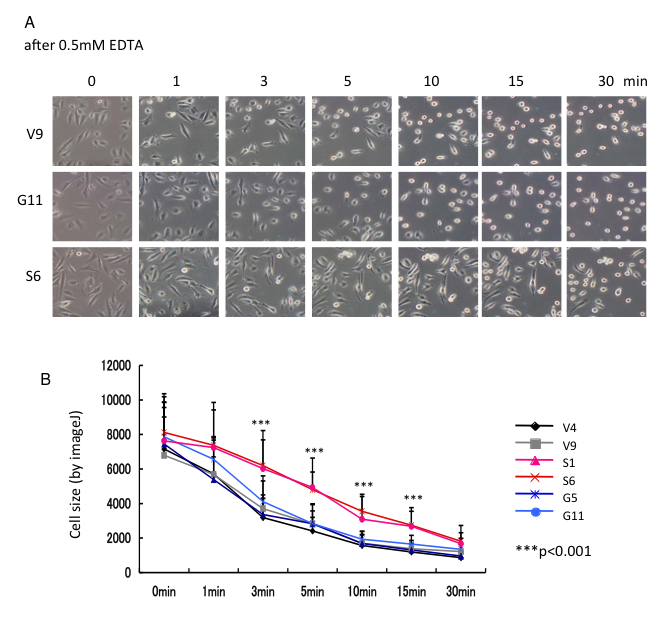

Supplement: S4 Fig — In usual experiments, GD2+ cells were highly resistant to detachment with EDTA treatment. Then, we examined wheather GD2+ cells are resistant to EDTA. At 3, 5, 10 and 15 min after EDTA treatment, detachment of cells were analyzed by morphology (A) and by counting detached cell number (B). GD2+ cells were resistant to detachment. These data suggested that GD2+ cells strongly adhere to plates in a cadherin-independent manner. (TIF) [file pone.0206881.s004.tif]

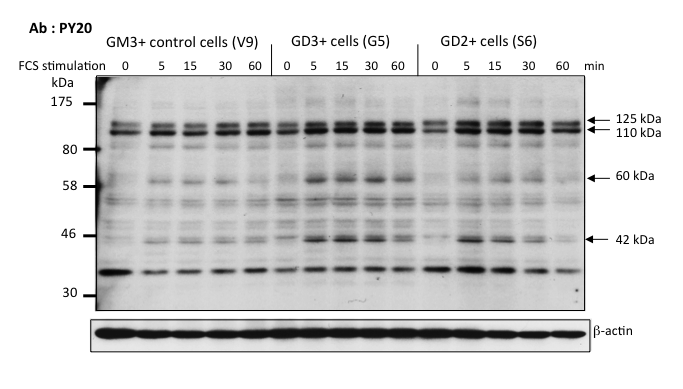

Supplement: S5 Fig — To analyze proteins involved in the cellular phenotypes of GD3+ and GD2+ cells, western immunoblotting with an anti-phosphotyrosine antibody (PY20) was performed using cell lysates prepared after FCS treatment. Cells were plated in dishes and serum-starved for 20 h before the treatment with FCS. (TIF) [file pone.0206881.s005.tif]
